# A Standard Scale to Measure Equine Keeper Status and the Effect of Metabolic Tendency on Gut Microbiome Structure

**DOI:** 10.3390/ani11071975

**Published:** 2021-07-01

**Authors:** Alexa C. B. Johnson, Amy S. Biddle

**Affiliations:** Department of Animal and Food Sciences, University of Delaware, Newark, DE 19716, USA; alexaj@udel.edu

**Keywords:** metabolism, keeper status, equine, microbiome

## Abstract

**Simple Summary:**

Horses with different metabolic tendencies are anecdotally referred to as “easy” or “hard” keepers. Easy keepers tend to gain weight easily while hard keepers require extra feed to maintain condition. Both easy and hard keeper horses carry a managerial and financial burden which can be a dissuading factor for horse shoppers. This research uses energy intake/need and body condition to develop a standard Equine Keeper Status Scale (EKSS) for assigning keeper status. The microbiome compositions based on EKSS assignments are then compared to explore microbiome differences based on metabolic tendencies of each group. The EKSS can be used by owners to accurately assess their horses’ metabolic tendencies and make improved feeding decisions to meet their horses’ needs. Understanding microbiome differences between easy, medium and hard keeper horses points to potential microbial roles in these metabolic tendencies.

**Abstract:**

Thriftiness in horses has been associated with more efficient nutrient harvesting in digestion, absorption and/or utilization, but the relative contribution of the gut microbiome to host metabolic tendency is not well understood. Recognizing the unreliability of owner reported assignment of keeper status, this research describes a novel tool for calculating whether a horse is an easy (EK) or hard (HK) keeper and then characterizes microbiome differences in these groups. The Equine Keeper Status Scale (EKSS) was developed and validated based on data gathered from 240 horses. Estimates of dietary energy intakes and requirements to achieve the optimal BCS score of 5 were used in EKSS assignments. Sixty percent of owners’ characterizations disagreed with EKSS identified keeper assignments. Equine fecal 16S rRNA profiles (*n* = 73) revealed differences in α and β diversities and taxa abundances based on EKSS assignments. EK communities had more Planctomycetes and fewer Euryarcheaota, Spirochaetes and Proteobacteria than HK indicating functional differences in nutrient harvesting between groups. Differences in the gut microbiomes of horses based on keeper assignment point to host/microbial interactions that may underlie some differences in metabolic tendency. The EKSS enables robust, repeatable determination of keeper status which can be used by researchers and horse owners.

## 1. Introduction

The definition of the equine keeper status is ambiguous and poorly characterized within the equine community. The current ideology behind the keeper status is the combination of equine body condition and how readily the animal maintains weight to determine how the horse should be fed [1]. Overweight horses that can easily maintain a Body Condition Score (BCS) (per the Henneke scale [2]) ≥ 6 are “easy keepers” (EK) and underweight horses that easily maintain a BCS ≤ 4 are “hard keepers” (HK) and both groups struggle to maintain or achieve an ideal BCS of 5. A third classification, “medium keepers” (MK) describes horses that can easily maintain a BCS = 5. Keeper status is applicable to all horses, independent of activity level, age, breed, gender and health status, and has been demonstrated in other species [3,4]. Animal owners use body condition as a key indicator of horse health and consider weight loss being a greater cause for concern (than weight gain) as it reflects more poorly on the quality of care [5]. Subsequently, owners may make inappropriate feed choices based on perceived negative connotations of owning an overweight/obese or underweight horse [6] or social pressure to provide their animal with treats and grain-based meals [7,8] regardless of the horse’s needs.

As a result of the ambiguity of equine keeper identification and the pressure of social biases, EK and HK horses are fed two drastically different diets. To reduce weight gain, EKs are often fed restricted and depleted diets [9] and to induce weight gain HKs are often fed extremely energy dense diets. Both of these feeding methods are antithetical to the anatomical design of the equine gut and can result in gut dysbiosis [10,11].

A normal “healthy” microbiome hosts a diverse microbial ecosystem that ferments the horse’s diet of primarily high-fiber plant material to meet as much as 70% of the horse’s energy requirements [12,13]. Equine body condition reflects the energetics and fermentative activities of the hindgut microbiome as well as each horse’s ability to utilize available nutrients. Even minor shifts in microbial populations in response to external parameters such as diet composition [11,14,15,16,17,18,19,20], abrupt dietary changes [17,21,22] and management practices [23,24] have been shown to impact the overall nutrition and energy profiles of the host. While it is acknowledged that management factors affect the microbiome, it is equally important to note that the inter-animal composition can vary greatly between individuals and yet still be “normal” for that individual [25]. It is difficult to determine the cause-and-effect relationship between the microbiome, the host and animal management choices; yet the dynamic relationship between the horse and its gut microbiome likely reflects individual capacities to harbor specific populations as well as host specific abilities to utilize available nutrients.

The first objective of this study is to present the Equine Keeper Status Scale (EKSS), a standardized scale to categorize equine metabolic tendency. The second objective of this study is to compare differences in microbiome structure based on EKSS versus owner reported keeper statuses (ORKS). We hypothesize that using the EKSS’s standardized and measurable assignments will enable greater resolution in taxonomic composition not evident with owner reported keeper statuses. Despite inter-horse variation due to diet and other host factors we present general trends revealed by EKSS groupings that indicates higher diversity in MK horses and less variability when compared to EK and HK horses. The EKSS is an easily applied tool that is accessible to the average horse owner and equine professionals such as equine nutritionists, veterinarians and researchers to assign accurate keeper status and make informed management decisions based on energy needs.

## 2. Materials and Methods

This experiment was approved by the University of Delaware Animal Care and Use Committee (#107R-2019-0).

### 2.1. Development of the Equine Keeper Status Scale

The EKSS is comprised of three equations and utilizes the relationships of equine body condition score (BCS), digestible energy (DE) maintenance requirements and DE intake to determine the horse’s keeper status. BCS is used to estimate the present condition of each horse with a score of 5 as the ideal benchmark. BCS is a veterinary standard measurement of how the animal distributes fat along the body [2] as body weight is a poor predictor of body condition scores in animals and humans [26]. DE was chosen since it is the recognized industry standard for evaluating equine nutrient requirements according to the National Research Council’s (NRC) consensus study report [27]. The NRC also recognizes DE as the most consistent and comparable metric for evaluating equine nutrient requirements because of the variability of dietary sources/composition that can stem from variability in animal feeding regimes due to animal purpose, geography, health issues, finances and accessibility to specific nutritional sources. The ability of the equine host to extract energy from feed, indicated by the difference between DE intake and requirement levels, serves as the central nutritional benchmark for the EKSS scale and presents a standardized approach to evaluate overall metabolic efficiency across various dietary sources of energy. Additionally, the EKSS equations are in agreement with the guidance provided by the NRC consensus report defining DE maintenance requirements as the amount of energy needed to prevent a change in the total energy contained in the body [27].

The EKSS was developed using horses of varying workload, age, sex, diet and breed. The EKSS is not developed for use in donkeys and mules as these equids have much better feed conversion ratios compared to horses and fat distribution is significantly different compared to horses [28]. The EKSS can be modified to managers’ preferences for the ideal BCS and DE adjustment rate. These modifications may be due to sport preferences for leanness (endurance racing), or reproductive preferences for thickness (broodmares) as well as health needs to adjust BCS scores safely for individual animal needs.

(1) 5−(A)=B

Equation (1) is the difference in BCS score from the ideal BCS score of 5. A is the horse’s current cumulative BCS. Cumulative BCS is determined by the Kohnke modification [29] of the Henneke BCS system where each region of the horse is scored individually and regions are averaged to obtain a cumulative BCS. B is the horse’s BCS difference from the ideal score (i.e., 5).

(2) $$\;B\times \left({\mathrm{DE}}_{M\;\left(\mathrm{Mcals}\right)}\times \;\Delta \mathrm{DE}\%\right)=C$$

Equation (2) calculates the megacalories required to achieve an ideal BCS score. DE_M(Mcals)_ is the individual animal’s digestible energy maintenance requirement in megacalories. The DE_M(Mcals)_ is calculated using the most accurate “Type” of animal according to the daily nutrient requirements of horses (NRC, Tables 16-1–16-5 [27]) and horse’s current body weight. ΔDE% is the digestible energy adjustment rate. For this study, we used a ΔDE% of 12.5% based on the recommendations outlined by the NRC [27]. The NRC [27] states that to change BCS by one-unit, digestible energy intake should be increased or decreased by 11–15% above or below the maintenance requirement. C is the change in megacalories required to achieve an ideal BCS score of 5.

(3) $$\frac{D+C}{{\mathrm{DE}}_{M(\mathrm{Mcal})}}\;\times \;100\;=\;{\mathrm{RDI}}_{5}\%$$

Equation (3) calculates the required dietary energy intake to achieve the ideal BCS score of 5. D is the horse’s current digestible energy intake (DEI) in megacalories. RDI_5_% is the recommended dietary intake percent to achieve a BCS of 5.

**Figure 1:**
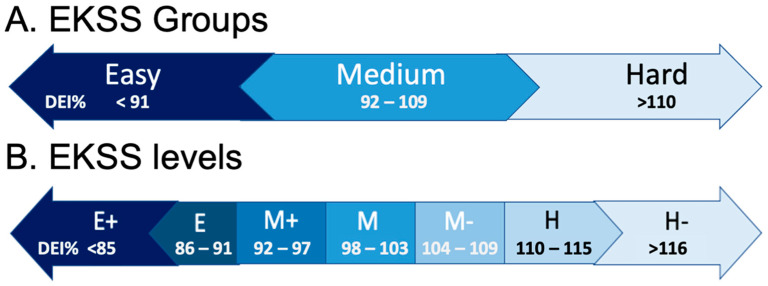
The EKSS offers two identification resolutions, The EKSS groups (**A**) and EKSS levels (**B**). The EKSS groups are offered to agree with the current usage of easy keeper (EK), medium keeper (MK) and hard keeper (HK) terminology but can be further defined to the EKSS level which proposes seven different keeper statuses. RDI_5_% ranges were determined by the recommendations listed by the NRC recommendation that a 10–15% change in DEI can change the BCS score by one point.

The EKSS offers two identification resolutions: EKSS groups (Figure 1A) and EKSS levels (Figure 1B). EKSS groups are assigned to correspond to the current usage of EK, MK and HK terminology. EKSS groups can be further divided into EKSS levels which propose seven different keeper statuses. RDI_5_% ranges for each EKSS group and level were determined by the recommendations outlined by the NRC specifying that a 10–15% increase or decrease in DEI is needed to change the BCS score by one unit.

### 2.2. Development and Testing of the EKSS

#### 2.2.1. Sampling Protocol

The EKSS was calculated for horses (*n* = 240) of mixed sexes, age, breed, diet and workload. Further descriptive data of the horses can be found in Table S1. Horses were recruited from June 2019 through November 2020 in the MidAtlantic Region. ORKS were obtained by verbal responses and owners’ ORKS decisions were not influenced by the research team. ORKS were obtained from the feeding manager of each farm (*n* = 16) who was considered ‘experienced’ with a minimum four years in the position. The feeding managers were responsible for 15 horses on average (min = 2, max = 53, s.d. ± 12). ORKS distribution by farm can be found Figure S1.

The EKSS was designed to be accessible for a wide audience ranging from the average horse owner to make better feeding management decisions and monitor animal weight to clinical researchers who need a repeatable, measurable and quantifiable method to identify animals for study enrollment. To most accurately replicate the conditions and data that the average horse owner would be capable and willing to collect, we used field-based techniques and commercial products that are readily available and easy to use.

Equine body weight was obtained using a weight tape (Coburn Horse & Pony weight tape (Whitewater, WI, USA)) at the heart-girth and was measured by the same researcher each time to reduce user error. The weight tape method was chosen as it is the preferred option for owners and veterinarians as a field-based method to measure weight [30]. Cumulative BCS was measured as the average of two independent and blinded scorers using the Kohnke modification [29] of the Henneke Body Condition Scoring system [2]. Simply, each body region (tailhead, crease down the back, crest of the neck, withers, ribs, and behind the shoulder) was scored on a scale of 1–9 and a cumulative BCS was obtained by averaging the regions. Cumulative BCS scores of the independent scorers were then averaged to obtain an overall average BCS. BCS scores were ranked as “obese” (BCS > 6), “normal” (BCS = 5–6), and “lean” (BCS < 5).

##### Diet Estimations

A commercial online equine nutrition calculator (www.FeedXL.com, accessed on 6 May 2019) was used to estimate dietary composition of individual equine diets. FeedXL was chosen because of its ease of use, commercial availability, extensive library of feed, pasture, and hay analyses, as well as the option to input personal forage analyses and ability to edit guaranteed analyses of feeds. It is highly unlikely that owners perform routine forage analyses, therefore, to capture the most likely extent and use of the EKSS we replicated methods the EKSS users would employ. If owners did have forage analyses available, these reports were obtained and manually imported to FeedXL. Grain and supplement feed tags were collected (if possible) for guaranteed analyses or were obtained online and imported or updated in the FeedXL feed library.

All daily offered feed sources (i.e., grains, supplements and hay) were individually weighed to obtain a comprehensive diet report for each horse. Any ad libitum hay or pasture was estimated by FeedXL based on a rate of intake given a range of time the horse had access to the ad libitum feed. The general rule that horses are able to ingest up to 2% of their bodyweight [27] was taken into account in cases where horses had 16+ hours of access to ad libitum feed and the ad libitum feed was calculated up to that 2% bodyweight threshold minus the intake amount from other offered feed sources (i.e., offered hay and grain). All horses had ad libitum access to water.

Equine activity level was determined by the work level described by the owners and classified as ‘no work’, ‘light’, ‘moderate’, ‘heavy’, and ‘very heavy’ by the research team as described by the NRC (Tables 1–10) [27]. In FeedXL, “Normal Keeper Status” was selected regardless of the ORKS assignment since changing “keeper status” within FeedXL altered the maintenance requirements. All maintenance requirements were double checked via manual calculations from the NRC (NRC, Tables 16-1–16-5 [27]). The overall average BCS was rounded to the nearest whole number for FeedXL, but it was determined that changing BCS score in FeedXL did not affect nutrient reports. Disease status was always set to “None” regardless of horse health status as reported by owners since this affected commentary suggestions from FeedXL and did not affect nutrient intake reports.

Hay quality offered to horses was ranked by the research team from the FeedXL choices of ‘Prime’, ‘Good’, ‘Average’, ‘Poor’, and ‘Weather damaged’. Hay qualities were assessed visually for color, smell, stem thickness, leafiness and presence of weeds, mold or sunburn. Pasture quality was ranked by the research team from the FeedXL choices of ‘Excellent’, ‘Good’, ‘Average’, and ‘Poor’. Pasture qualities were assessed visually for grass composition, color, presence of weeds, patchiness and over-grazed qualities (grass swards < 1” in height).

#### 2.2.2. Statistical Analysis

All data was evaluated in R statistical software [31]. Equine BCS and diet compositions were analyzed using Kruskal–Wallis tests and pairwise Wilcoxon rank sum tests with post hoc testing using the Benjamini–Hochberg false discovery rate adjustment correction. Statistical significance was determined at (*p* ≤ 0.05) and a tendency towards significance at (0.05 ≤ *p* ≥ 0.10). Equine estimated total body fat (eTBF) was calculated using the equation reported by Dugdale et al. [32]: $e^{TBF\;}=0.006+e^{1.56\times BCS}$.

### 2.3. Microbiome Surveying of EKSS Horses

#### 2.3.1. Sampling Protocol

Subsequent to the sampling protocol described above, a fecal sample was collected from each horse (*n* = 73, farm; *n* = 9) for 16S profiling. Farms 3, 4, 5, and 12 were chosen for fecal collection since they contained a large number of animals and consistent feeding regimes. Horses were then selected from additional farms to adjust sampling groups to achieve an even group distribution. For enrollment, horses were required to have no prior or active gut health issues and no antibiotics, non-steroidal anti-inflammatory drugs, vaccinations or anthelmintics within 180 days prior to fecal collections. Descriptive data on these horses can be found in Tables S1 and S2. Immediately following defecation, the fecal pile was broken open to reveal feces at the center of the fecal pile that would have little to minimal environmental contamination. A sterile spoon was then used to collect ~4.0 g of feces and placed into a sterile 5 mL tube containing 1 mL of DNA/RNA shield (Zymo Research, Tustin, CA, USA) and shaken to distribute the solution throughout the sample. Fecal samples were stored at −20 °C for a maximum of 7 days until DNA extraction could be performed.

#### 2.3.2. DNA Extraction and Sequencing

DNA was extracted from fecal samples using the QIAGEN QIAmp Powerfecal DNA Isolation Kit (Germantown, MD, USA) according to manufacturer’s instructions. DNA was tested for quantity and quality using a Qubit 3.0 fluorometer (ThermoFisher Scientific, Waltham, MA, USA) and Nanodrop spectrophotometer (ThermoFisher Scientific, Waltham, MA, USA). The V4-V5 region of 16S rRNA gene was amplified using universal primers (515yF 3′-GTGYCAGCMGCCGCGGTAA-5′/926pfR 3′-CCGYCAATTYMTTTRAGTTT-5′) and sequenced using normalized DNA pools and dual-barcoded Illumina MiSeq library preparation (RTL Genomics, Lubbock, TX, USA). Primer choice was based on established Earth Microbiome Protocols [33].

#### 2.3.3. Bioinformatic and Statistical Analysis

Microbial data processing and statistics were performed using QIIME2 (Quantitative Insights Into Microbial Ecology, v. 2020.8) and following the workflow described in the “Moving Pictures” tutorial (https://docs.qiime2.org/2021.4/tutorials/moving-pictures/, accessed on 19 March 2021). Taxonomic assignments were made against the SILVA_132_99_16S database [34] trained to the 515F/926R primer set. QIIME2 plug-ins q2-composition (ANCOM differential abundance testing) was used for statistical analysis (QIIME2 v. 2020.8). The ANCOM F-statistic is a measure of the effect size difference for a particular species between study groups and the W-statistic is the strength of the ANCOM test for the tested number of species.

OTU tables were exported to R [31] for further statistical analysis and visualizations with the ‘phyloseq’ package [35]. OTU tables were normalized to the median sequencing depth (4149.5 bp) and rarefied to the lowest sequence count (2240 reads) per sample for beta diversity analysis. Alpha diversity measures (Shannon and Observed) were calculated with the normalized OTU table and tested with ANOVA and Tukey’s multiple pairwise tests. The Shannon index estimates sample richness and evenness, and the Observed index estimates the true number of genera within samples.

Beta diversity measures, weighted Unifrac, nonmetric multidimensional scaling (NMDS) and Bray–Curtis Dissimilarity principal coordinate analysis (PCoA) plots were calculated with normalized and rarefied OTU tables. Bray–Curtis Dissimilarity matrix measures community composition using an abundance-based approach to calculate dissimilarity between all samples while the weighted Unifrac method uses phylogenetic relatedness in addition to abundance counts. Statistical analysis of the distance matrices was calculated with PERMANOVA (permutational multivariate analysis of variance) of the Adonis function in the ‘vegan’ R package [36].

Bacterial abundances were statistically analyzed using Kruskal–Wallis and Wilcoxon Rank Sum Tests and significance was determined at *p* < 0.05 and a tendency towards significance at (0.05 ≤ *p* ≥ 0.10). Pairwise Spearman rank correlations (r) were calculated with R statistical software and significance was determined at (0.3 ≤ r ≥ −0.3). The r is fairly significant at (≥0.3–<0.5), moderate with (≥0.5–<0.7), strong with (≥0.7–<0.9), and substantial with (≥0.9–1.0).

## 3. Results

### 3.1. Development and Testing of the EKSS

#### 3.1.1. Keeper Status Distribution

**Figure 2:**
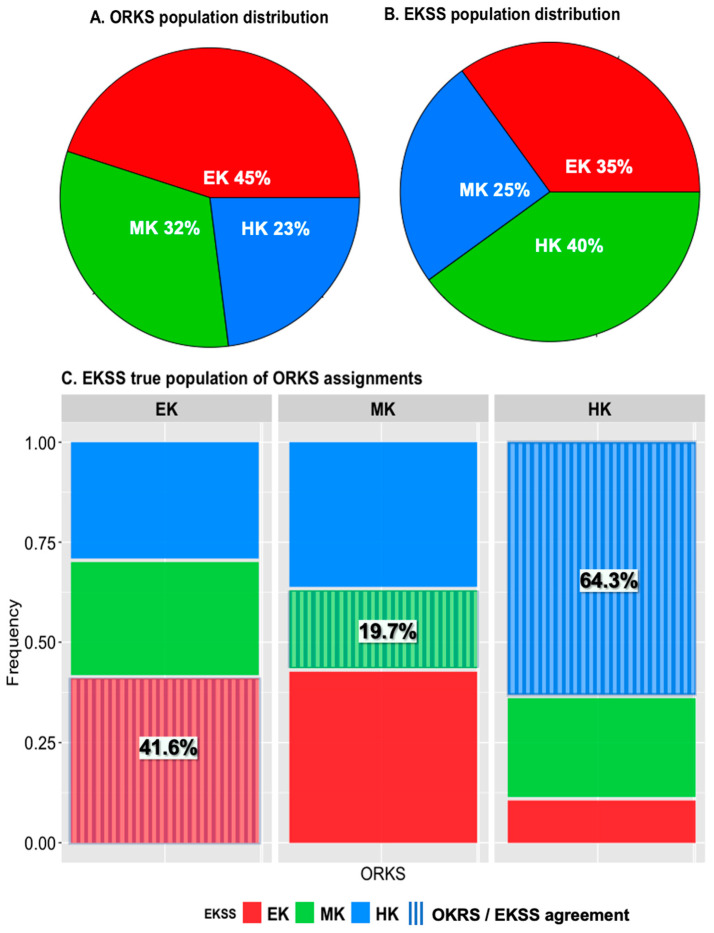
Distribution of keeper statuses in ORKS and EKSS. In all three plots, easy keeper (EK) = red, medium keeper (MK) = green and hard keeper (HK) = blue. When ORKS assignments are used (**A**) the EK makes up the largest proportion of the population at 45% followed by MK and HK. When the population is re-evaluated using EKSS assignments (**B**) the HK makes up the largest proportion of the population. To determine how often ORKS and EKSS assignments were in agreement, a mosaic plot was used (**C**). Stripes represent agreement between ORKS and EKSS statuses. This demonstrates that owners had the least difficulty in identifying HK and had the most difficulty identifying MK.

Owners reported their horse’s keeper status to be: 45% EK, 32% MK and 23% HK (Figure 2A). The same horses were re-assigned EKSS keeper statuses and the population rates were found to be 35% EK, 25% MK and 40% HK (Table 1, Figure 2B). Of the EKSS levels, 58% of the population was determined to be E+ (24%) and H- (34%) (Table 1). According to BCS ranks, the distribution was 39% obese, 53% normal and 8% lean (Table 1).

When the horses were reassigned with EKSS assignments only 40% of EKSS assignments matched with the original ORKS assignment given. 41.6%, 19.7% and 64.3% of ORKS EK, MK and HK were identified in agreement with the EKSS (Figure 2C).

#### 3.1.2. Equine Body Characteristics and Dietary Composition

Body composition means (BCS and estimated total body fat (eTBF%)) differed between EKSS keeper statuses (<0.001, <0.001), respectively (Table 2). EKSS statuses showed that forage intake did not differ between keeper statuses but grain intake, total intake %, DEI% and CPI% differed between EK, MK and HK groups (Table 2). Total intake % indicated that EKSS HK were eating at a higher rate than 2% of their bodyweight which appeared to be due to the provision of grains as forage intakes did not differ between keeper statuses (Table 2).

**Figure 3:**
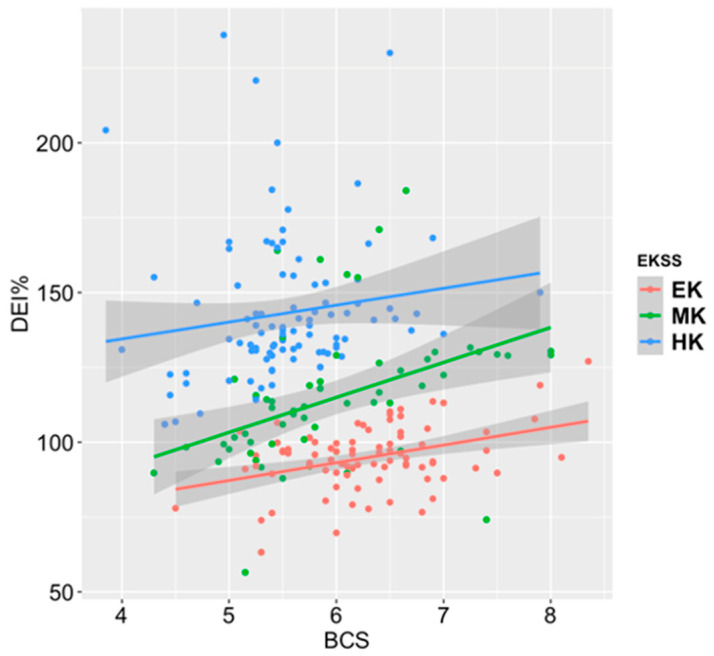
Linear models of DEI% and BCS for the three keeper groups. Shading indicates 95% confidence intervals. Easy keeper (EK) = red, Medium keeper (MK) = green, Hard keeper (HK) = blue.

To determine if horses were overweight due to an overabundance of feed or underweight due to an insufficient amount of feed, linear models of DEI% and BCS were plotted with EKSS assignments (Figure 3). When the horses were assigned with EKSS assignments, the model shows a positive slope between DEI% and BCS. Forty-eight (20%) of the animals were obese due to an overabundance of feed (DEI ≥ 110% and BCS ≥ 6) and only 2 animals (0.8%) were shown to be lean due to being underfed (DEI% ≤ 91% and BCS ≤ 5).

### 3.2. Microbiome Survey of EKSS Horses

The total feature frequency across all samples (*n* = 73) was 21,548 and an average feature frequency of 7692 per sample (±s.d = 3525; range = 2256–15,841; median = 6739). Average read length was 410.31 (±s.d = 3.21; range = 263–478). Read counts following the denoising, quality filtering, and chimera check steps as implemented in QIIME2 are reported on Table S5.

#### 3.2.1. Alpha and Beta Diversity

Alpha diversity refers to the number of species (richness) and how they are distributed (evenness) within each sample. Observed species estimates the true number of bacteria and the Shannon index measures evenness and richness. Shannon and Observed species did not differ between ORKS groups (*p* > 0.05) but did differ between EKSS groups (Shannon, *p* = 0.06; Observed, *p* = 0.04) (Table 3). EKSS MK had the highest alpha diversity values for both Shannon and Observed species indices and EKSS EK had the lowest values. Tukey’s all-pair comparison tests found the difference between EKSS EK and MK alpha diversity measures to be the most different (*p* < 0.05) with EKSS HK falling in between.

**Figure 4:**
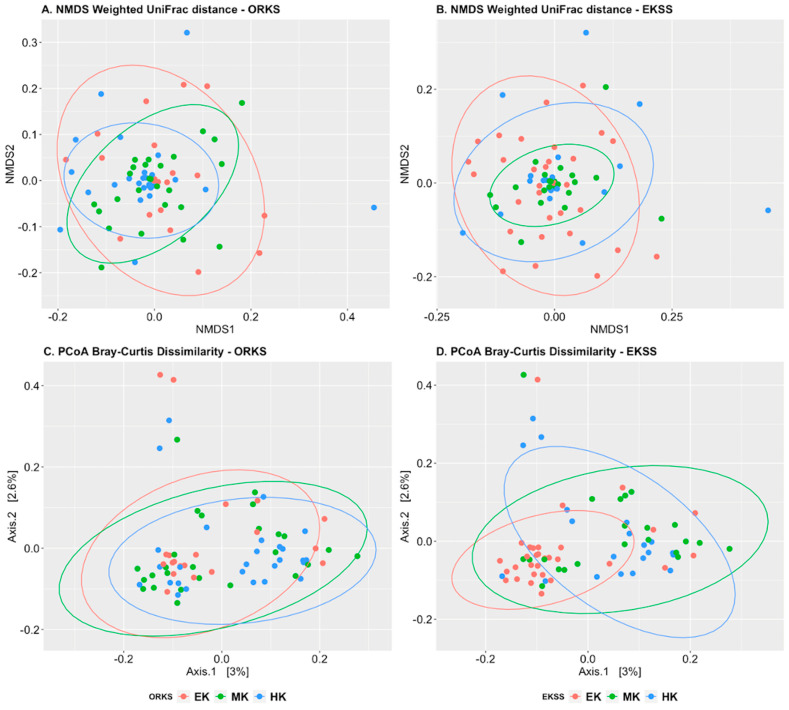
Beta diversity plots of owner reported keeper status (ORKS) and equine keeper status scale (EKSS). (**A**) Weighted Unifrac NMDS plot of ORKS. (**B**) Weighted Unifrac NMDS plot of EKSS. (**C**) Principal coordinate analysis (PCoA) plots of Bray–Curtis Dissimilarity plots of ORKS. (**D**) Principal coordinate analysis (PCoA) plots of Bray–Curtis Dissimilarity plots of EKSS Ellipsoids demonstrated at 95% confidence interval around each group. In both ORKS and EKSS plots easy keeper (EK) = red, medium keeper (MK) = green and hard keeper (HK) = blue.

Weighted Unifrac and Bray–Curtis plots (Figure 4) both showed a difference in ORKS centroids (adonis, *p* = 0.07 (Figure 4A) and 0.03 (Figure 4C), respectively) but it was not due to ORKS assignments (betadisper, *p* = 0.58 and 0.48). However, EKSS showed a difference in group centroids (adonis, *p* = 0.02 (Figure 4B) and 0.002 (Figure 4D), respectively) and it was due to EKSS assignments (betadisper, *p* = 0.03 and 0.001). The non-significance betadisper values for the ORKS indicated that another factor was the cause behind the different centroids.

#### 3.2.2. Relative Abundance, Differential Abundance Testing, Spearman Correlations

**Figure 5:**
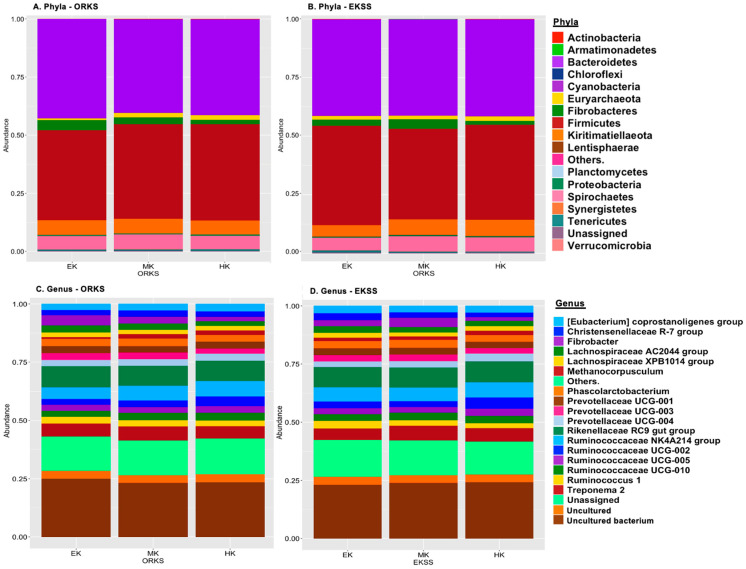
Relative abundance of the bacteria at the phyla level and the top 20 genera level bacteria present at rates >1%. Bacteria present at rates lower than 1% are represented by “Others.”. (**A**) Relative abundance of the top phyla in owner reported keeper statuses (ORKS). (**B**) Relative abundance at the phyla level in equine keeper status scale (EKSS) (**C**) Relative abundance of the top genera in ORKS. (**D**) Relative abundance of the top genera in EKSS.

The relative abundances of bacteria at phyla and genera levels present at rates >1% of ORKS and EKSS are presented in Figure 5.

ANCOM differential abundance testing was performed at all bacterial levels and detected four phyla and one class to differ between EKSS groups (Table 4). ANCOM testing was also performed on BCS rank assignments, farm and age category and found 1, 13, and 12 taxa to be differentially abundant between groups. No taxa were determined to be differentially abundant between gender, breed category or ORKS groups (Table S3).

Spearman correlations identified bacteria to be correlated with all ORKS and EKSS statuses except for ORKS MK. EKSS EK was negatively correlated with ORKS HK (−0.37), EKSS MK was negatively correlated with ORKS EK (−0.38) and EKSS HK was positively correlated with ORKS HK (0.42) (Table S2). The positive spearman correlation between ORKS HK and EKSS HK demonstrates strong agreement between ORKS and EKSS which suggests that owners had the least difficulty at identifying HK status as also shown in Figure 2C. The negative correlations between ORKS EK-EKSS HK (−0.37) and EKSS MK-ORKS EK (−0.38) revealed the highest rates of disagreement in keeper status assignment between EKSS and ORKS (Table S2).

Planctomycetes, Clostridiales, Bacteroidales UCG 001, Peptococcaceae, Succinivibrionaceae Mycoplasmataceae, *Clostridiales*, and *Ruminococcaceae* and *Succinivibrionaceae uncultured* were found two be positively correlated one EKSS status and negatively correlated with another EKSS status (Table 5). Correlations were identified with all EKSS groups, ORKS EK and ORKS HK (Table 5, Table S2).

## 4. Discussion

### 4.1. Assigning Metabolic Tendency Using the EKSS

#### 4.1.1. Keeper Status Distribution

The EKSS tool was designed to be easy for horse owners, veterinarians and researchers to learn and use with minimal technical skill, equipment, or cost. EKSS groupings maintain the current verbiage of the lay equine community (EK, MK, HK) and EKSS levels provide greater resolution and precision.

To the authors’ knowledge there is only one published report of keeper status population statistics [1]. Robin et al. [1] surveyed 792 owners in Great Britain and found that 62.9% of ponies and 35.2% of horses to be ‘good doers’/EK. This study stated that the ‘good doer’ animal was a common view in the equine industry and required less feed to maintain optimum body condition [1,38]. They further stated that the owner’s perception of how readily an animal maintains weight likely influenced feeding management and that owners justified high BCS based on the belief that ‘good doers’ easily maintain or gain weight on little feed [1]. We observed that owners reported 45% of the population to be EK and the EKSS assigned 35% of the population as EK (the comparable metabolic tendency to ‘good doers’). With no comparable reported population rates of MK and HK found in the literature, we report the first population rates for these animals with EKSS assignments (25% MK and 40% HK).

Overall, only 40% of ORKS agreed with EKSS assignments. The greatest agreement between ORKS and EKSS was found in HK identification (64.3%), followed by EK (41.6%). The least amount of agreement was found in MK identification (19.7%). These results may demonstrate that there is a heightened response of owners to identify leaner animals and a higher tolerance for over conditioned animals. This higher tolerance or inability to recognize over conditioned animals may stem from a higher level of acceptance and rates of obesity in sedentary and pleasure horses compared to performance horses due to their decreased energy expenditure rates and over- or maintained provision rates of energy dense feeds [1,39]. Show ring competing animals (judged on breed characteristics and performance) have also been trending towards obesity as an over conditioned animal appears to score better during competition [40,41,42].

A study performed by Thatcher et al. [43] in horses (*n* = 300) in Virginia during the summer of 2006, found that 51% of horses were over conditioned or obese. The results of the current study did not observe as high a rate of obesity even when the BCS categories of Thatcher et al. [43] were used (24% of the population, data not shown). Studies performed in Great Britain [1], Scotland [26], and North Carolina [44] report over conditioned/obese horses, all using slightly different BCS methods, in ranges from 31–48%. Although the experimental design, equine enrollment, determination of BCS, exercise, dietary intake, and geographic proximity between Thatcher et al. [43] and this study were similar, we report very different obesity rates. It is possible that obesity rates in the MidAtlantic region have declined since reported by Thatcher et al. [43] in 2006 in response to increasing awareness regarding the dangers of equine obesity and the availability of new feed options for weight management.

Equine metabolic efficiency and its relationship to keeper status has been suggested to be a genetic trait [43,45,46] with over conditioning/obesity to be found at higher rates in the Rocky Mountain Horse, Tennessee Walking Horse, Quarter Horse, Warm Blood and Mixed Breed horses compared to the Thoroughbred [43]. In the current study we also observed higher rates of obesity in the Warm Blood (38%), Cold Blood (76%) and Pony breed (73%) categories compared to the Hot Blood category (17%) (data not shown). The results of this study agree that certain breeds have a genetic tendency towards over conditioned or leaner phenotypes, but this study also observed that owners reported all three keeper statuses within each breed category and within farms in some cases. This points towards individual animal variability for metabolic tendencies to gain, maintain or lose weight easily within breed categories. We did not attempt to determine the genetic conditions that regulate metabolic efficiencies, but we highlighted that there are animals that are exceptions to breed stereotyped lean/obese predispositions.

#### 4.1.2. Equine BCS and Dietary Composition

The EKSS observed significantly different feeding patterns in EK, MK and HK animals with EK animals having lower forage and grain intakes, total intake %, DEI% and CPI% with a linear increase across all categories from EK towards the HK. This demonstrated the value of the EKSS in assisting owners in formulating rations based on measured metabolic tendency, measured DE values, and recommendations toward customizable body condition goals.

### 4.2. Gut Microbiome Difference Based on EKSS

Due to the observational study design utilized in this study the horses enrolled were not controlled for diet or other animal characteristics or management. However, all horses were provided access to some combination of pasture and grain and some were supplemented with additional hay. Diet is a proven driver of the microbial consortia [11,14,16], but large inter-individual variations [25,47,48] are also known attributes of microbiome surveying. Efforts were made in this study to include a large sample size (*n* = 73) from numerous farms to reduce the influence of the diet on our microbial interpretations. We utilized 16S microbial surveying as complimentary evidence of the robustness of the EKSS in identifying general trends within the EKSS keeper statuses compared to ORKS assignments.

#### 4.2.1. Alpha and Beta Diversity

General ecology theories indicate that increased alpha and beta diversity has a stabilizing effect for gut microbiome communities [49] and that reduced diversity is an indicator of gastrointestinal disease [22,50]. In this study, the EKSS MKs had the highest diversity. Thus we hypothesized that MK communities may be more stable than EK and HK microbiome communities according to these theories.

Whereas in the ORKS group the HK had the highest alpha diversity and EK had the lowest which may be a reflection of feeding management techniques between EK and HK horses. Willette et al. [22] found that horses that were withheld from feed had decreased alpha diversity indices compared to horses that had ad libitum access to feed. This study observed the same alpha diversity trend of Willete et al. [22] in EK animals that are also fed less feed to induce a negative energy balance when compared to MK and HK feeding management.

This study found that beta diversity measures of ORKS groups had large overlaps and thus little distinction between keeper status. When beta diversity was reanalyzed with EKSS assignments, weighted Unifrac plots showed a tighter clustering of MK which indicated more phylogenetic similarity of the commonly present bacterial populations when compared to the EK and HK that were more dissimilar based on the larger ellipsoids. The increased spread in the weighted Unifrac plots indicated that there were more phylogenetically dissimilar and rarer bacteria present. The PCoA Bray–Curtis plot showed a high intraspecific aggregation of bacterial abundance in the EK group and that beta diversity abundance varied more within the MK and HK.

Diversity trends observed in the EKSS groups indicated that MK gut microbiomes were more diverse compared to that of the EK and HK horses. Differences in microbial diversity indices were resolvable with EKSS and not with ORKS thus supporting our first hypothesis that the EKSS is more discriminating at identifying keeper status than ORKS, and our second hypothesis that the MK is a more stable microbiome compared to EK and HK.

#### 4.2.2. Relative Abundance, Differential Abundance Testing, Spearman Correlations

Equine microbiome compositions are extremely sensitive to dietary changes and the differences reported here were likely due to the different feeding strategies used to feed the EK and HK horses. Similar to other studies [11,51,52,53] the equine gut microbiome was dominated by Bacteroidetes and Firmicutes. Studies have observed higher ratios of Firmicutes/Bacteroidetes (F/B) in obese horses followed by lean and ‘normal’ animals, respectively [52]. The current study found that the F/B ratio was not different between EK and HK (both 1.12/1) or MK (1.01/1). These results may differ from previous reports because the EKSS utilizes the relationship of BCS and DE to evaluate metabolic tendency and assign keeper status rather than relying on BCS alone. While the EK horses tended to be over conditioned (BCS ≥ 6) and HK horses tended to be underconditioned (BCS ≤ 4) a variety of BCS scores were present in EK and HK categories based on the amount of energy required to maintain the animal’s BCS. At the same time, the MK category had very little variety with a BCS = 5–6.

Planctomycetes have been previously identified to be associated with obese horses [52] and are suspected of being opportunistic pathogens as a member of the *PVC* superphylum along with Verrucomicrobia, and Chlamydia [54]. Culture experiments have shown that these are slow growing bacteria that grow best in low-nutrient-poor media [54] and rely on symbiotic relationships with other phyla such as Alphaproteobacteria, Bacteroidetes and Verrucomicrobia for nutrients [54,55,56,57]. It has been hypothesized that obese hosts have an increased capacity to absorb nutrients [58,59] which would create a less nutrient rich gut environment that is ideal for Planctomycetes proliferation. Results of the current study are in agreement with previous literature regarding Planctomycetes since this group was found to be in the highest abundance (0.337%) in the most over conditioned keeper status category (EK) (BCS = 6.31).

Euryarchaeota contains the members of the methanogenic archaea. Archaea can make up to 0.3–3.0% of the rumen microbiome in cattle [60,61] with the majority of the population being methanogenic. One study found that archaea was not included in the core microbiome of equids which means that low abundance archaea populations are likely to vary greatly between animals. Of the seven archaea genera found in horses, the methanogenic *Methanocorpusculum* and *Methanobrevibacter* made up to 44.7–51.2% of all archaeal 16S rRNA sequences per animal [62]. Methanogens are known to metabolize H_2_ and CO_2_ to produce methane [63]. Interspecies competition for H_2_ in the anaerobic gut system can cost ~5–9% loss of energy to the host [64]. When the H_2_ is sequestered for methanogenesis it is lost to other anaerobic fermentation pathways such as acetogenesis, which produces acetate as a readily available energy source for the host [65]. This study found the highest abundance of Euryarchaeota in the HK (2.02%) (Table 4) and that the methanogenic family (Methanomethylophilaceae) and genera (*Candidatus Methanomethylophilus*) were negatively correlated with EK (Table 5). Although the archaea are not included in reported equid core microbiomes, the small change to energy balance due to the presence or absence of methanogenic archaea in EKSS equid sub-groups may have significant impacts on energy availability and body weight for the horse over time [58].

Spirochaetes have been characterized from gut environments to serve important fibrolytic functions, specifically for hemicellulose [66]. This group has been reported in the equine gut at abundances from 0.5–3.5% [11,51,53] and appears to be associated with weight management in horses. In a study looking at the gastrointestinal impacts of weight loss in horses, Spirochaetes were significantly greater in the low weight-loss group compared to the high weight loss group [10]. Biddle et al. [52] found that Spirochaetaceae; *Treponema* to be differentially abundant between the Obese and Normal BCS groups. In the current study, EK and HK Spirochaetes are both found in greater abundance compared to the values reported elsewhere [10,11,51,53]. The availability of hemicellulose for both groups via high forage intake (EK’s feeding management to induce negative-energy balance for weight loss) and/or retention (HK’s difficulty to maintain weight and low BCS) may explain the greater abundances of Spirochaetes in these animals.

Proteobacteria are a diverse bacterial group thought to play important roles in global carbon, nitrogen and sulfur cycling in both soil and gut communities [67,68]. While Proteobacteria are important for nutrient cycling, blooms of the class Gammaproteobacteria have been associated with inflammation, microbial dysbiosis, and colic [48,69]. During normal anaerobic fermentation, Gammaproteobacteria are less able to thrive because of the lack of molecular oxygen or NO_3_^−^ for oxidative phosphorylation [70]. However, when inflammation occurs, these terminal electron acceptors, provided by the host via denitrification, become readily available for the Gammaproteobacteria and allow them to outgrow and outcompete the other anaerobes in the gut, resulting in a chronic cycle of inflammation [70,71,72]. At the phyla level, Proteobacteria was in the highest abundance in the MK (1.088%) which may point toward their contribution to healthy nutrient cycling within this more stable microbiome. However, the Gammaproteobacteria were found to be in the highest abundance in the HK (0.272%) which may be due to the high oil and high starch diets commonly fed to HKs to increase weight [11]. Moreover, equine diets that contain high levels of starch have also been associated with dysbiosis resulting in inflammatory illnesses like lactic acidosis, colic, and laminitis [73]. This study did not observe clinical signs of inflammation in horses at the time of the collection and horses were excluded if they had had prior gut health issues. Therefore, we cannot conclude that HK animals were experiencing low levels of gut inflammation from this study and controlled experiments would be necessary to determine this.

With the exception of Spirochaetes, differentially abundant bacteria identified by ANCOM in EKSS assignments were also identified by Spearman correlations supporting the ANCOM findings that significantly identified taxa can be identified by multiple testing methods. One bacterial lineage (Proteobacteria, Gammaproteobacteria, Aeromondales, Succinivibrionaceae, *Succinivibrionaceae*, *uncultured*) was found to be positively correlated with EKSS HK at the order, family and genus level, and negatively correlated with EKSS EK at the phyla level and genus level. In cattle, *Succinovibrio* were found to be more abundant on the high starch diet [74,75] and were identified as an important component of the gut microbiome of bees consuming a high starch diet [76]. The positive correlation of *Succinivibrionaceae*, *uncultured* with HK and negative correlations with EK further supports the hypotheses that *Succinivibrio* has a role in starch metabolism within the gut microbiome.

Taxonomic approaches to identify different species in microbial communities have been widely used, but only a fraction of the microbial population in the horse gut has been well described with many understudied groups [77,78,79]. Therefore, it has been a topic of debate about which taxonomic resolution provides the best trade-off between taxonomic detection and interpretation [80]. For example, the phyla level offers high community coverage but low community resolution while the genera level offers low community coverage but high community resolution. Our poor understanding of these organisms, particularly at the lowest taxonomic levels stems from the fact that they are difficult to culture or have complex behaviors within the microbiome and the host [79]. Salis et al. [80] concluded that broad ecological generalizations could be made at the phyla level [80,81], and that the responses of the order level were generally representative of the responses of the genera and species levels to stressors. The current study conducted tests (ANCOM (Table 4 and Table S3) and Spearman correlations (Table 5 and Table S2)) at all taxonomic levels to represent the complex responses of bacteria that may be hidden at higher taxonomic resolutions and present these findings as an opportunity for future research to investigate these taxa within EKSS phenotypes.

Taxonomic approaches towards characterizing the microbiome are additionally limited in the ability to accurately capture the complex intrinsic microbial interactions that occur between the host and community including symbiosis, cross-feeding, antagonism, competition, and predation [81]. It is plausible that through microbial plasticity and functional redundancy of the microbiome significantly different taxonomic compositions may not necessarily translate to different functionalities and vice versa [53,58,79]. The role of functional redundancy in obesity-associated microbiomes has been described [53,58] which may provide insight into differences between the EK, MK and HK animals but further study would be needed to measure or predict microbial function.

Surveys of gut microbial communities are inherently limited by technological limitations and inter-individual variabilities due to management factors, host physiology, and the metabolic plasticity and functional redundancy of the microbiome. Despite this variability, this study reports statistically significant microbial patterns that appear to be associated with EKSS keeper statuses. The microbial taxa found to be associated with keeper statuses are reasonable and are validated by prior papers. These microbial patterns serve as complimentary evidence of the relative discriminating ability of the EKSS as these patterns were only resolvable with the application of the EKSS tool and not found by owner reported assignments.

## 5. Conclusions

The EKSS is a valuable and easy tool for horse owners, feeding managers, veterinarians in the field, and equine research and development teams to accurately assign keeper status and evaluate the relationships between feeding management, body condition, and energy intake. The EKSS is an easy-to-use measure that will alleviate confusion and inform feeding management for all horses.

Distinctions found in the observational study of the gut microbiome structure based on EKSS assignments suggest differences in microbial diversities and phylogenetic community relatedness within and between keeper statuses. It is unclear with this study population if these differences are the consequence of feeding methods for EK (feed restriction) and HK (overfeeding of starch) or the selection of specific community members by host conditions. The lower alpha and beta diversities of the EKSS EK and HK groups may indicate that these gut communities are less stable and have a greater sensitivity to dysbiosis than the MK group. However, a controlled study to reduce diet, management and equine factors is needed to validate microbiome differences associated with EKSS EK, MK and HK statuses.

## Figures and Tables

**Table 1:** Distribution of keeper statuses.

	EKSS Horses (*n* = 240)	Fecal Sampling (*n* = 73)
**EKSS Group ^1^**		
EK	35 (84)	42 (31)
MK	25 (60)	30 (22)
HK	40 (96)	27 (20)
**EKSS Level ^2^**		
E+	24 (57)	30 (22)
E	11 (27)	12 (9)
M+	9 (22)	11 (8)
M	9 (21)	11 (8)
M-	7 (17)	8 (6)
H	6 (14)	7 (5)
H-	34 (82)	21 (15)
**BCS Rank ^3^**		
Lean	8 (18)	7 (5)
Normal	53 (128)	62 (45)
Obese	39 (94)	32 (23)
**EKSS and ORKS Keeper Agreement ^4^**		
Yes	40 (95)	47 (34)
No	60 (145)	53 (39)

Values are reported in percentage of the population and values in parenthesis are counts. ^1^ Equine Keeper Status Scale Groups (see Figure 1). ^2^ EKSS levels are described by Figure 1. ^3^ BCS Rank: Lean (BCS < 5), Normal (BCS = 5–6), Obese (BCS > 6). ^4^ Comparison of ORKS and EKSS assignments.

**Table 2:** Diet composition means of EKSS assignments (*n* = 240).

	EK	MK	HK	*p*-Value
BCS	6.31 ^a^ (0.70)	5.95 ^b^ (0.84)	5.52 ^c^ (0.65)	<0.001
eTBF% ^1^	9.84 ^a^ (1.09)	9.29 ^b^ (1.31)	8.61 ^c^ (1.01)	<0.001
Forage intake, kg	8.05 ^a^ (1.79)	8.19 ^a^ (2.45)	8.15 ^a^ (2.62)	<0.001
Grain intake, kg	1.63 ^c^ (1.36)	2.38 ^b^ (1.87)	3.36 ^a^ (2.96)	<0.001
Total intake, % ^2^	2.05 ^b^ (0.19)	2.02 ^b^ (0.23)	2.33 ^a^ (0.53)	<0.001
DEI, % ^3^	94.92 ^c^ (10.66)	114.39 ^b^ (23.76)	143.03 ^a^ (24.49)	<0.001
CPI, % ^4^	167.91 ^c^ (35.24)	197.34 ^b^ (33.68)	225.96 ^a^ (33.20)	<0.001

Values represent means with standard deviation in parenthesis. *p*-values were determined by Kruskal–Wallis tests between EKSS statuses. ^a–c^ Superscripts within the row indicates *p* < 0.05 by pairwise Wilcoxon rank sum tests with post hoc testing using the Benjamini–Hochberg false discovery rate adjustment correction. ^1^: eTBF% = estimated total body fat (equation from Dugdale et al. [32]). Forage and grain intakes are represented in as-fed kilograms to demonstrate how much bulk feed horses are provided. ^2^: Total intake % = the total feed intake (kg) relative to the animal’s bodyweight (kg). ^3^: DEI% = digestible energy intake percent relative to the animal’s digestible energy maintenance requirements. ^4^: CPI% = crude protein intake percent relative to the animal’s crude protein maintenance requirements.

**Table 3:** Alpha diversity measures Shannon and Observed effects on ORKS compared to EKSS statuses.

	ORKS				EKSS			
	EK	MK	HK	*p*-Value	EK	MK	HK	*p*-Value
Shannon	5.28 ^a^	5.43 ^a^	5.50 ^a^	0.34	5.30 ^a^	5.62 ^b^	5.37 ^ab^	0.06
Observed sp.	351 ^a^	390 ^a^	432 ^a^	0.28	353 ^a^	469 ^b^	378 ^ab^	0.04

Differing superscripts within the row of owner reported keeper statuses (ORKS) and equine keeper status scale (EKSS), respectively, demonstrates significance between keeper statuses as determined by (*p* < 0.05) with Tukey’s all-pair comparison method.

**Table 4:** Relative abundances of the ANCOM identified taxa to be differentially abundant between EKSS groups.

	EKSS Abundance			ANCOM	
	EK	MK	HK	W ^1^	clr ^2^
Planctomycetes ^3^	0.337	0.002	0.098	3	5.22
Euryarchaeota ^3^	1.611	1.452	2.020	1	3.27
Spirochaetes ^3^	4.595	0.055	5.402	1	2.88
Proteobacteria ^3^	0.646	1.088	0.960	1	2.55
Gammaproteobacteria ^4^	0.063	0.001	0.272	18	8.44

^1^ the W-statistic is the strength of the ANCOM test for the tested number of species. ^2^ the clr F-statistic is a measure of the effect size difference for a particular species between the study groups. ANCOM determines significance by plotting the F-statistic on the *x*-axis by the W-statistic on the *y*-axis [37]. ^3^ Phyla taxonomic level. ^4^ Class taxonomic level.

**Table 5:** Spearman correlations.

	EKSS		
	EK	MK	HK
**Phyla**			
Planctomycetes	0.33		−0.30
Proteobacteria	−0.30		
**Class**			
Gammaproteobacteria ^2^			−0.36
Bacteroidia ^3^			−0.32
**Order**			
Rickettsiales ^2^			0.32
Aeromondales ^2^			0.36
Izimaplasmatales ^4^		0.41	
Clostridiales ^5^	0.41		−0.31
**Family**			
Methanomethylophilaceae ^1^	−0.32		
Succinivibrionaceae ^2^			0.36
Burkholderiaceae ^2^	−0.31		
Bacteroidales RF16 group ^3^	−0.31		
Bacteroidales UCG 001 ^3^	0.39	−0.32	
Muribaculaceae ^3^	0.31		
Mycoplasmataceae ^4^	−0.32	0.30	
Peptococcaceae ^5^		0.31	−0.34
Gastranaerophilales, uncultured rumen bacterium ^6^		0.32	
**Genera**			
*Candidatus methanomethylophilus* ^1^	−0.33		
*Succinivibrionaceae, uncultured* ^2^	−0.31		0.32
*Bacteroidia* ^3^			−0.32
*Bacteroidales, F082* ^3^			−0.33
*Clostridiales; f_; g_* ^5^	0.41		−0.31
*Eubacterium oxidoreducens group* ^5^	0.39		
*Lachnospiraceae UCG 008* ^5^			−0.38
*Marvinbryantia* ^5^	−0.31		
*Ruminococcaceae NK4A214 group* ^5^	−0.30		

^1^ Belonging to Euryarchaeota phylum. ^2^ Belonging to Proteobacteria phylum. ^3^ Belonging to Bacteroidetes phylum. ^4^ Belonging to Tenericutes phylum. ^5^ Belonging to Firmicutes phylum. ^6^ Belonging to Cyanobacteria phylum. Spearman rank correlations (r) were determined with R statistical software and significance was determined at (−0.3 ≤ r ≥ 0.3). The r is fairly significant at (≥0.3–<0.5), moderate with (≥0.5–<0.7), strong with (≥0.7–<0.9), and substantial with (≥0.9–1.0). Red boxes indicate a negative correlation and green indicates a positive correlation. Yellow rows indicate taxa significantly correlated with two EKSS groups.

## Data Availability

The datasets generated during this study have been deposited in the NCBI Sequence Read Archive: https://www.ncbi.nlm.nih.gov/sra, accessed on 14 March 2021, Bioproject: PRJNA715971. Accession numbers can be found in the Supplemental Materials (Table S4).

## Supplementary Materials

The following are available online at https://www.mdpi.com/article/10.3390/ani11071975/s1, Table S1: Description of equine subjects, Table S2: ORKS spearman correlations, Table S3: ANCOM results of BCS, Farm, Sex, Age category, Breed category and ORKS, Table S4: NCBI accession numbers, Table S5: Sequencing statistics.
