# Supplementary material for: A Standard Scale to Measure Equine Keeper Status and the Effect of Metabolic Tendency on Gut Microbiome Structure
Source: Animals (Basel). 2021 Jul 1;11(7):1975. doi: 10.3390/ani11071975 (PMC8300108; doi:10.3390/ani11071975)
Supplement: Supplementary file 1 [file animals-11-01975-s001.zip › animals-1232250-supplementary.pdf]

animals

Supplementary Materials

A Standard Scale to Measure Equine Keeper Status and the Effect of Metabolic Tendency on Gut Microbiome Structure

Alexa C.B. Johnson and Amy S. Biddle *

Department of Animal and Food Sciences, University of Delaware, Newark, DE 19716, USA; alexaj@udel.edu
* Correspondence: asbiddle@udel.edu

Citation: Johnson, A.C.B.; Biddle, A.S. A Standard Scale to Measure Equine Keeper Status and the Effect of Metabolic Tendency on Gut Microbiome Structure. *Animals* **2021**, *11*, 1975. https://doi.org/10.3390/ani11071975

Academic Editor: Marcio Costa

Received: 7 May 2021
Accepted: 29 June 2021
Published: 1 July 2021

Publisher's Note: MDPI stays neutral with regard to jurisdictional claims in published maps and institutional affiliations.

Copyright: © 2021 by the authors. Licensee MDPI, Basel, Switzerland. This article is an open access article distributed under the terms and conditions of the Creative Commons Attribution (CC BY) license (http://creativecommons.org/licenses/by/4.0/).

Table S1. Description of equine subjects.

	**EKSS Horses (*n* = 240)**	**Fecal Sampling (*n* = 73)**
Age [1]		
Young	29 (70)	23 (17)
Mature	56 (134)	56 (41)
Old	15 (36)	21 (15)
Sex		
Mare	43 (103)	34 (25)
Gelding	57 (137)	66 (48)
Breed [2]		
Cold blood	9 (21)	16 (12)
Warm blood	57 (137)	45 (33)
Hot blood	25 (60)	29 (21)
Pony	9 (22)	10 (7)
Season		
Spring	20 (49)	67 (49)
Summer	47 (113)	15 (11)
Fall	30 (71)	8 (6)
Winter	3 (7)	10 (7)
Farm [3]		
n	(16)	(7) [a]

Values are reported in percentage of the population and values in parenthesis are counts. [1] – Age is categorized into three divisions: Young (010 years), Mature (1120 years), Old (21+ years). [2] – Breed is categorized into three divisions: Cold blood (European descent, heavyboned horses including Drafts and Draft crosses), Warm blood (horses crossed between Cold and Hot blood horses), Hot blood (Middle East descent, lightboned horses including Thoroughbreds and Arabians). [3] – The number of farms in the study. [a]: Farms included in the fecal sampling protocol are: Farm 2, 5, 10, 9, 11, 12, 14 and 15.

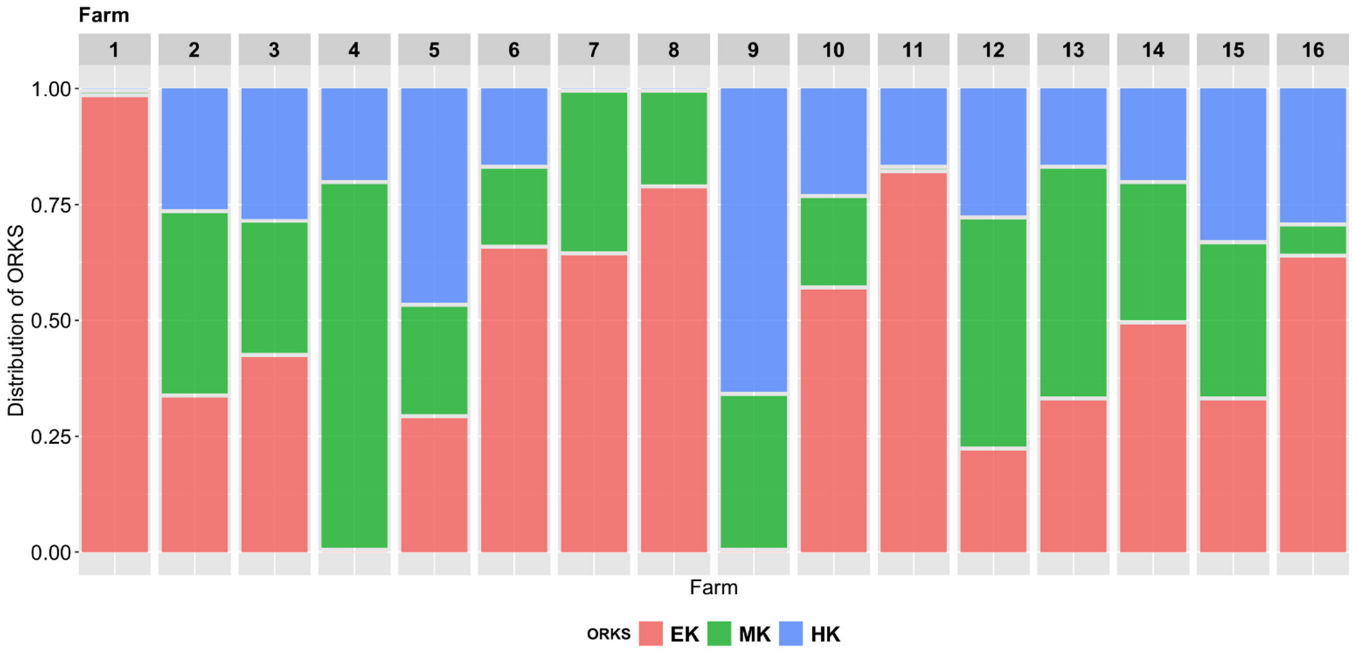

Figure S1. Distribution of ORKS by farm. Each bar represents a farm and the colors represent the proportion of ORKS EK (pink), ORKS MK (Green) and ORKS HK (Blue). Farms reported all three keeper statuses as being present in their herd aside from 6 farms (Farms 1, 4, 7, 8, 9, and 11). Farm 1: $n = 2$. Backyard barn. Farm 2: $n = 53$. Therapeutics/Riding School. Farm 3: $n = 14$. Pasture animals. Farm 4: $n = 10$. Competitive Hunter Jumper Barn (all levels). Farm 5: $n = 17$. Riding school. Farm 6: $n = 18$. College equestrian team herd. Farm 7: $n = 23$. Riding school. Farm 8: $n = 5$. Backyard barn. Farm 9: $n = 3$. Backyard barn. Farm 10: $n = 26$. Breeding barn. Farm 11: $n = 6$. Backyard barn Farm 12: $n = 18$. Boarding barn. Farm 13: $n = 12$. Boarding barn. Farm 14: $n = 10$. Competitive ThreeDay Eventing barn (all levels). Farm 15: $n = 6$. College research herd. Farm 16: $n = 17$. Pasture animals.

Table S2. Spearman correlations.

	ORKS		
	EK	MK	HK
Keeper Status			
EKSS, EK			0.37
EKSS, MK	0.38		
EKSS, HK			0.42
Phyla			
Euryarchaeota	0.32		
Order			
Aeromondales [1]			0.37
Family			
Succinivibrionaceae [1]			0.37
Izimaplasmatales, uncultured bacterium [3]			0.32
Ruminococcaceae [4]	0.33		
Genera			
Succinivibrionaceae, uncultured [1]			0.39
F082, Bacteroidales bacterium Bact 22 [2]	0.30		
F082, Bacteroidia bacterium canine oral taxon 187 [2]			0.32
Lachnospiraceae AC2044 group [4]			0.35
*Ruminococcaceae; g_* [4]	0.45		0.41

[1] Belonging to Proteobacteria phylum. [2] Belonging to Bacteroidetes phylum. [3] Belonging to Tenericutes phylum. [4] Belonging to Firmicutes phylum. Spearman rank correlations (r) were determined with R statistical software and significance was determined at (0.3 ≤ r ≥ 0.3). The r is fairly significant at (≥0.3<0.5), moderate with (≥0.5<0.7), strong with (≥0.7<0.9), and substantial with (≥0.91.0). Red boxes indicate a negative correlation and green indicates a positive correlation.

Table S3. ANCOM results of BCS, Farm, Sex, Age category, Breed category and ORKS.

	**ANCOM – W score**
BCS	
k__Bacteria;p__Spirochaetes;c__MVP15;o__uncultured rumen bacterium	19
FARM	
k__Bacteria;p__Kiritimatiellaeota;c__Kiritimatiellae	27
k__Bacteria;p__Proteobacteria;c__Gammaproteobacteria	26
k__Bacteria;p__;c__	26
k__Bacteria;p__Firmicutes;c__	21
k__Bacteria;p__Proteobacteria;c__Gammaproteobacteria;o__Aeromonadales	76
k__Bacteria;p__Kiritimatiellaeota;c__Kiritimatiellae;o__WCHB141	69
k__Bacteria;p__;c__;o__	69
k__Bacteria;p__Proteobacteria;c__Gammaproteobacteria;o__Aeromonadales;f__Succinivibrionaceae	144
k__Bacteria;p__;c__;o__;f__	136
k__Bacteria;p__Firmicutes;c__Clostridia;o__Clostridiales;f__Ruminococcaceae;g__Ruminococcaceae UCG010	269
k__Bacteria;p__;c__;o__;f__;g__	258
k__Bacteria;p__Bacteroidetes;c__Bacteroidia;o__Bacteroidales;f__F082;g__	242
k__Bacteria;p__Proteobacteria;c__Gammaproteobacteria;o__Aeromonadales;f__Succinivibrionaceae;g__uncultured	240
AGE CATEGORY	
k__Bacteria;p__	5
k__Unassigned;p__	1
k__Bacteria;p__Bacteroidetes	1
k__Bacteria;p__Epsilonbacteraeota	1
k__Bacteria;p__;c__	5
k__Bacteria;p__Spirochaetes;c__MVP15	2
k__Bacteria;p__Cyanobacteria;c__Oxyphotobacteria	2
k__Unassigned;p__;c__	1
k__Bacteria;p__Actinobacteria;c__	1
k__Bacteria;p__Planctomycetes;c__Phycisphaerae	1
k__Bacteria;p__Fibrobacteres;c__Fibrobacteria	1
k__Bacteria;p__Proteobacteria;c__	1

Analysis was conducted at the phyla, class, order, family and genera taxonomic levels. No bacteria were identified by ANCOM to be differentially abundant by gender, breed and ORKS categories and are not shown.

Table S4. NCBI Accession Numbers.

Accession	Sample Name	BioProject
SAMN18389454	EMP205	PRJNA715971
SAMN18389455	EMP206	PRJNA715971
SAMN18389456	EMP207	PRJNA715971
SAMN18389457	EMP208	PRJNA715971
SAMN18389458	EMP209	PRJNA715971
SAMN18389459	EMP210	PRJNA715971
SAMN18389460	EMP211	PRJNA715971
SAMN18389461	EMP212	PRJNA715971
SAMN18389462	EMP213	PRJNA715971
SAMN18389463	EMP214	PRJNA715971
SAMN18389464	EMP215	PRJNA715971
SAMN18389465	EMP216	PRJNA715971
SAMN18389466	EMP217	PRJNA715971
SAMN18389467	EMP218	PRJNA715971
SAMN18389468	EMP219	PRJNA715971
SAMN18389469	EMP220	PRJNA715971
SAMN18389470	EMP221	PRJNA715971
SAMN18389471	EMP222	PRJNA715971
SAMN18389472	EMP223	PRJNA715971
SAMN18389473	EMP224	PRJNA715971
SAMN18389474	EMP225	PRJNA715971
SAMN18389475	EMP226	PRJNA715971
SAMN18389476	EMP227	PRJNA715971
SAMN18389477	EMP228	PRJNA715971
SAMN18389478	EMP229	PRJNA715971
SAMN18389479	EMP230	PRJNA715971
SAMN18389480	EMP231	PRJNA715971
SAMN18389481	EMP232	PRJNA715971
SAMN18389482	EMP233	PRJNA715971
SAMN18389598	EMP125	PRJNA715971
SAMN18389601	EMP128	PRJNA715971
SAMN18389603	EMP130	PRJNA715971
SAMN18389604	EMP131	PRJNA715971
SAMN18389605	EMP132	PRJNA715971
SAMN18389606	EMP133	PRJNA715971
SAMN18389607	EMP134	PRJNA715971
SAMN18389608	EMP135	PRJNA715971
SAMN18389609	EMP136	PRJNA715971
SAMN18389610	EMP137	PRJNA715971
SAMN18389611	EMP138	PRJNA715971
SAMN18389612	EMP139	PRJNA715971
SAMN18389613	EMP140	PRJNA715971
SAMN18389614	EMP141	PRJNA715971
SAMN18389615	EMP142	PRJNA715971
SAMN18389616	EMP143	PRJNA715971
SAMN18389617	EMP144	PRJNA715971
SAMN18389618	EMP145	PRJNA715971
SAMN18389619	EMP146	PRJNA715971
SAMN18389620	EMP147	PRJNA715971
SAMN18389621	EMP148	PRJNA715971
SAMN18389622	EMP149	PRJNA715971
SAMN18389623	EMP150	PRJNA715971
SAMN18389624	EMP151	PRJNA715971
SAMN18389625	EMP152	PRJNA715971

SAMN18389627	EMP154	PRJNA715971
SAMN18389628	EMP155	PRJNA715971
SAMN18389629	EMP156	PRJNA715971
SAMN18389630	EMP157	PRJNA715971
SAMN18389632	EMP161	PRJNA715971
SAMN18389633	EMP163	PRJNA715971
SAMN18389639	EMP169	PRJNA715971
SAMN18389640	EMP171	PRJNA715971
SAMN18389648	EMP179	PRJNA715971
SAMN18389649	EMP182	PRJNA715971
SAMN18389650	EMP183	PRJNA715971
SAMN18389652	EMP185	PRJNA715971
SAMN18389653	EMP186	PRJNA715971
SAMN18389654	EMP187	PRJNA715971
SAMN18389655	EMP188	PRJNA715971
SAMN18389656	EMP189	PRJNA715971
SAMN18389657	EMP190	PRJNA715971
SAMN18389658	EMP203	PRJNA715971
SAMN18389659	EMP204	PRJNA715971

The datasets generated during this study have been deposited in the NCBI Sequence Read Archive: https://www.ncbi.nlm.nih.gov/sra, Bioproject: PRJNA715971.

Table S5. Sequencing statistics.

Sample ID	Input	Filtered	Percentage of Input Passed filter	Denoised	Merged	Percentage of Input Merged	Nonchimeric	Percentage of Input Nonchimeric
EMP125	75023	37885	50.5	34024	12145	16.19	6053	8.07
EMP128	73977	37258	50.36	31559	8750	11.83	4934	6.67
EMP130	97080	51337	52.88	44233	13715	14.13	6925	7.13
EMP131	93595	46926	50.14	41234	13141	14.04	6534	6.98
EMP132	70348	34120	48.5	28542	8408	11.95	3981	5.66
EMP133	82032	41774	50.92	37437	14701	17.92	6348	7.74
EMP134	55189	28725	52.05	23141	4700	8.52	2540	4.6
EMP135	70416	35503	50.42	29412	9390	13.34	5546	7.88
EMP136	58460	29579	50.6	25703	7766	13.28	4286	7.33
EMP137	44172	20848	47.2	17152	4820	10.91	2892	6.55
EMP138	94809	47472	50.07	40064	10472	11.05	5654	5.96
EMP139	102940	50784	49.33	43158	12423	12.07	6150	5.97
EMP140	68290	31241	45.75	26557	9249	13.54	4108	6.02
EMP141	101379	50651	49.96	44514	14498	14.3	7734	7.63
EMP142	89306	42583	47.68	33469	7156	8.01	3240	3.63
EMP143	54634	14791	27.07	10989	2303	4.22	1455	2.66
EMP144	55270	27252	49.31	22777	5826	10.54	4142	7.49
EMP145	51972	25382	48.84	20751	5987	11.52	2792	5.37
EMP146	83331	42004	50.41	34485	7730	9.28	4533	5.44
EMP147	93837	46383	49.43	40108	12536	13.36	5934	6.32
EMP148	68750	31234	45.43	26201	7757	11.28	3961	5.76
EMP149	92212	44488	48.25	40081	16359	17.74	7496	8.13
EMP150	90470	42486	46.96	33950	5976	6.61	3715	4.11
EMP151	63799	30060	47.12	22874	4478	7.02	2948	4.62
EMP152	41185	16231	39.41	12873	2828	6.87	1502	3.65
EMP154	94524	47711	50.48	40284	12208	12.92	5536	5.86
EMP155	81970	70399	85.88	62034	0	0	0	0
EMP156	75802	33669	44.42	27227	8137	10.73	4035	5.32
EMP157	102625	50899	49.6	45969	17757	17.3	8521	8.3
EMP161	84250	41362	49.09	34093	7094	8.42	4372	5.19
EMP163	97153	43389	44.66	35932	8457	8.7	5831	6
EMP169	74340	35994	48.42	30042	8610	11.58	3938	5.3
EMP171	102228	50575	49.47	44842	17382	17	6538	6.4
EMP179	84317	38892	46.13	32933	9364	11.11	5628	6.67
EMP182	21037	11840	56.28	8795	2992	14.22	2022	9.61
EMP183	21224	11793	55.56	9375	2895	13.64	2218	10.45
EMP185	21636	12071	55.79	9199	3449	15.94	1180	5.45
EMP186	21052	11508	54.66	9132	2700	12.83	2012	9.56
EMP187	20607	11523	55.92	9021	2607	12.65	1624	7.88
EMP188	20842	11695	56.11	9750	4579	21.97	2297	11.02
EMP189	21796	11871	54.46	9470	3426	15.72	1749	8.02
EMP190	24868	13672	54.98	11305	3357	13.5	2001	8.05
EMP203	15181	8414	55.42	6444	2265	14.92	1411	9.29
EMP204	24175	12361	51.13	9825	3965	16.4	2386	9.87
EMP180	54171	47169	87.07	41758	16623	30.69	8422	15.55
EMP201	19516	17702	90.71	15361	5681	29.11	2284	11.7
EMP202	31675	27644	87.27	24256	10687	33.74	4865	15.36
EMP205	22523	19431	86.27	16413	6261	27.8	4216	18.72
EMP206	54535	46855	85.92	41585	17985	32.98	8274	15.17
EMP207	32877	28395	86.37	25250	10611	32.27	5249	15.97
EMP208	37129	33110	89.18	29654	12979	34.96	5765	15.53
EMP209	46269	40322	87.15	35784	13178	28.48	5237	11.32

EMP210	33553	29500	87.92	25993	9987	29.76	4021	11.98
EMP211	55269	47434	85.82	43150	17381	31.45	7255	13.13
EMP212	56608	48807	86.22	44200	20422	36.08	8151	14.4
EMP213	50252	43025	85.62	36150	9787	19.48	4918	9.79
EMP214	45894	38591	84.09	33506	13388	29.17	4969	10.83
EMP215	50750	43328	85.38	37776	14930	29.42	7569	14.91
EMP216	45073	39353	87.31	33947	11446	25.39	6224	13.81
EMP217	32169	28334	88.08	25023	9496	29.52	4582	14.24
EMP218	31883	28326	88.84	24042	7885	24.73	5375	16.86
EMP219	30223	27126	89.75	24071	9505	31.45	5077	16.8
EMP220	36945	29756	80.54	25793	9841	26.64	5506	14.9
EMP221	41613	36509	87.73	31329	11605	27.89	6920	16.63
EMP222	49935	43261	86.63	38121	13672	27.38	7163	14.34
EMP223	43014	37537	87.27	32667	11700	27.2	6246	14.52
EMP224	35224	32067	91.04	28842	12599	35.77	5370	15.25
EMP225	34013	30007	88.22	25651	8305	24.42	5017	14.75
EMP226	32022	28591	89.29	25180	8492	26.52	4509	14.08
EMP227	49455	43261	87.48	38546	15079	30.49	7509	15.18
EMP228	30968	26113	84.32	22704	8615	27.82	3858	12.46
EMP229	55062	47453	86.18	42125	15945	28.96	7343	13.34
EMP230	30917	27678	89.52	24210	6713	21.71	3894	12.6
EMP231	37729	33844	89.7	30301	10165	26.94	5928	15.71
EMP232	47337	41686	88.06	37581	16441	34.73	6706	14.17
EMP233	37518	33158	88.38	29433	12182	32.47	4585	12.22
EMP281	35562	30730	86.41	26427	10497	29.52	6126	17.23
EMP282	41769	36371	87.08	32533	13772	32.97	8112	19.42
EMP283	33313	29728	89.24	26104	10522	31.59	5418	16.26
EMP284	31648	24068	76.05	21443	8119	25.65	4704	14.86

Read counts and percentages for sequencing data following the dada2 amplicon merging, chimera and quality filtering steps.
